# Patient reported outcomes and recruitment rates following the introduction of principled patient information leaflets (PrinciPILs): Protocol for a meta-analysis

**DOI:** 10.3310/nihropenres.13420.1

**Published:** 2023-05-26

**Authors:** Jeremy Howick, Martina Svobodova, Shaun Treweek, Nina Jacob, Katie Gillies, Jennifer Bostock, Peter Bower, Adrian Edwards, Kerenza Hood

**Affiliations:** 1 Centre for Trials Research (CTR), College of Biomedical and Life Sciences, Cardiff University, Cardiff, Wales, UK; 2 Health Services Research Unit, University of Aberdeen, Aberdeen, Scotland, UK; 3 Division of Population Health, Health Services Research & Primary Care, The University of Manchester, Manchester, England, UK; 4 Division of Population Medicine, School of Medicine, Cardiff University, Cardiff, Wales, UK

**Keywords:** Communication, harms, nocebo, placebo, research ethics, meta-analysis, harms, adverse events, recruitment

## Abstract

**Background:**

The way potential benefits and harms of trial interventions are shared within patient information leaflets (PILs) varies widely and may cause unnecessary harms (“nocebo effects”). The aim of this meta-analysis will be to evaluate the influence on recruitment rates and early effects on patient reported adverse events of principled patient information leaflets (PrinciPILs) compared with standard PILs.

**Methods:**

Eligible studies will include those that report the effects on recruitment and patient reported adverse events of PrinciPILs compared to standard PILs. We will include in this meta-analysis all the standard PILs in studies within trials (SWATs) of PrinciPILs that were developed as part of the Medical Research Council (MRC) funded PrinciPIL project. By publishing this as a living meta-analysis, we will allow the meta-analysis to be updated with future SWATs of PrinciPILs. We will use the Cochrane Risk of Bias tool to evaluate the risk of bias for each outcome. We will report the total number of studies and participants analysed and the characteristics of included studies (including details of intervention, comparators, outcomes). For dichotomous data, we will calculate the risk difference and the risk ratio (RR) and 95% confidence intervals (CIs). For continuous outcomes we will use weighted mean differences with 95% CIs or standardized mean differences with 95% CIs. We will investigate heterogeneity by visually inspecting the forest plot and by considering the I ^2^ test result. We will assess the certainty warranted for each outcome using the Grading of Recommendations Assessment Development and Evaluation (GRADE). Ethics approval is not applicable since no original data will be collected. The results will be disseminated through peer-reviewed publication and conference presentations.

**Discussion:**

We will discuss the limitations of the meta-analysis including study risk of bias, inconsistency, heterogeneity, and imprecision. A general interpretation of the results and important implications will be provided.

## Plain language summary

People who take part in randomised trials need to understand the risks as well as the benefits of taking part. Most ‘patient information leaflets’ (PILs) that describe trial treatments include information about harms. Yet only some PILs contain information about potential benefits. This variation is confusing. Also, the over-emphasis on harms can cause “nocebo” effects, which are the harms caused by expecting something bad to happen. To solve these problems, we have developed seven principles that ensure that information about potential benefits and harms in PILs is balanced and consistent. We will now compare PILs that have been developed according to our principles (we call these ‘PrinciPILs’) with PILs that have not been developed with our principles. We will test whether PrinciPILs reduce nocebo effects and improve trial recruitment. Here we have described our plans to test the effect of PrinciPILs in a few trials.

## Introduction

### Rationale

Recent research has identified several problems with the way in which information about potential benefits and harms of trial treatments are presented within patient information leaflets (PILs). A review of 33 PILs from trials registered with the International Standard Randomised Controlled Trials Number (ISRCTN) clinical trial registry found that the way in which this information is presented varies widely, with some PILs not mentioning potential benefits at all ^1^. Relatedly, an overview of systematic reviews including over 250,000 trial participants given placebo treatments suggests that reported harms are increased by the way in which information about potential harms is presented ^2^. Presenting potential benefits and harms in an unbalanced way can also adversely affect trial recruitment ^3^. The way in which potential trial treatment benefits and harms are currently presented within PILs also raises a number of under-explored ethical issues. Specifically, emphasizing the need to share information about harms is needed for patients to provide informed consent, thus respecting the principle of autonomy. However, if the way in which this information is shared causes avoidable harm, this violates the ethical principle of non-maleficence (to do no harm) ^4^. Meanwhile, current Health Research Authority (HRA) guidance on describing potential benefits and harms is brief and does not appear to be applied consistently ^1^.

The Medical Research Council (MRC) in the UK funded a project to help solve the problem with variation in way in which information about potential benefits of trial interventions is shared within PILs, and avoidable harm caused by this variation ^5, 6^. As part of the project, a range of stakeholders (including patient representatives, medico-legal experts, and trial researchers) developed principles that could be used to present information about potential benefits and harms within PILs in a more consistent way. The principles have been used to adapt PILs from five host clinical trials (‘standard PILs’). We call the adapted PILs ‘Principled Patient Information Leaflets (PrinciPILs)’. Having the principles can reduce variability and provide guidance to help those who design PILs (and ethics committees who evaluate them).

In addition to being intrinsically valuable for reducing variability and harmonising the way PILs present information about potential benefits and harms of trial interventions, PrinciPILs are hypothesized to improve recruitment rates, reduce some reported harms, and improve quality of life ^4^. To test this hypothesis, the PrinciPILs will be compared with standard PILs in studies within trials (SWATs). However, individual SWATs will not be powered to detect changes in recruitment rates or early outcomes. Also, single SWATs do not enable comparison and generalisability of results of SWATs across different types of trials, populations, protocols, processes, and outcomes ^7^.

### Objectives

To synthesize the influence on recruitment rates and early outcomes of PrinciPILs as compared with standard PILs.

## Methods

This protocol was reported following the Preferred Reporting Items for Systematic Review and Meta-Analysis Protocols (PRISMA-P) guidelines ^8^. The meta-analysis will be conducted and reported following the Preferred Reporting Items for Systematic Reviews and Meta-Analyses (PRISMA) guidelines ^9^.

### Patient and Public Involvement

A patient and public involvement (PPI) representative (JB) was involved in acquiring the funding for this study, question development, research design, and background research. The same PPI representative is involved in our ongoing active dissemination plan for this study.

### Rationale for the use of a living method

A meta-analysis of a small number of SWATs may demonstrate a difference in the main outcomes. However, additional SWATs are likely to be required for the pooled comparison to be adequately powered. In addition, the effect of PrinciPILs in different contexts, diseases populations, and outcomes is likely to vary ^10^. Future SWATs of PrinciPILs are therefore likely to add relevant information about the effects of PrinciPILs. This meta-analysis is therefore designed to facilitate the inclusion of future SWATs of PrinciPILs.

### Eligibility criteria

Any SWAT comparing PrinciPILs with standard PILs is eligible for this meta-analysis ^11^.

***Population.*** We will not restrict the SWATs by population, and the trial populations of the SWATs varied widely.

***Intervention.*** The intervention for the included trials will be PrinciPILs.

***Comparators.*** The comparators for the studies will be standard PILs. These will be the PILs produced by the research teams of the host trials. We will summarize the differences between the PrinciPILs and standard PILs in a table.

***Outcomes.*** As agreed with the host trials, we will collect data on recruitment rates and early outcomes (within 3 months). We collected any early outcome that the host trial collected, and we anticipate that these will include data related to quality of life, pain, patient satisfaction, and retention.

***Timing.*** At recruitment and up to 3 months post-recruitment.

***Setting.*** There will be no restrictions by type of setting.

***Language.*** We anticipate that all trials will be reported in English, however, we will not place a language restriction on the PILs.

### Information sources

We will include all the SWATs of PrinciPILs that were developed as part of the MRC funded PrinciPIL project (as yet to be determined) in this meta-analysis. By publishing our study as a living meta-analysis, it may also be updated with future SWATs of PrinciPILs. We have included the protocol for SWATs of PrinciPILs on the Northern Ireland Trials Methodology Repository so that future researchers can conduct SWATs of PrinciPILs ^11^.

### Study records

***Data collection process.*** Using standardized pre-piloted forms (Microsoft Excel) and an instruction manual, two reviewers will input data independently with discrepancies resolved in discussion with a third reviewer. We will contact host study authors to resolve any uncertainties.

### Data items

We will report the name of the experimental and control interventions, a full description of both the intervention and control interventions, (where available) patient characteristics (average age, gender, ethnicity, symptoms), trial design, trial size, duration of follow-up, type and source of financial support.

### Outcomes and prioritization

We will report differences between the intervention (PrinciPIL) and control (standard PIL) groups. This will include differences in recruitment rates and all available early outcomes (gathered within 3 months of randomisation). We anticipate that these outcomes will include patient reported adverse events, quality of life, mortality, and trial recruitment rates. Since it can be difficult to distinguish between adverse events and illness symptoms, we will include patient reported illness symptoms as adverse events.

### Risk of bias in individual studies

We will use the Cochrane Risk of Bias tool (Rob2) to evaluate the risk of bias for each outcome. This tool includes five domains: bias arising from randomisation process, bias due to deviations from intended interventions, bias due to missing outcome data, bias in measurement of outcome, bias in selection of the reported result. In addition, an additional domain is available for cluster randomised trials; bias arising from identification or recruitment of individual participants within clusters. The risk of bias will be related to the implementation of the SWAT. Two reviewers will independently assess the risk of bias, with discrepancies being resolved by discussion with a senior reviewer (JH) if necessary.

### Effect measures

We will calculate differences for all outcomes between the intervention and control. For dichotomous data, we will calculate the risk difference and the risk ratio (RR) and 95% confidence intervals (CIs). For continuous outcomes we will use weighted mean differences with 95% CIs or standardized mean differences with 95% CIs.

### Data synthesis

We will start with a narrative synthesis of the results in the text and tables that summarizes the study characteristics and results.

Because the interventions and control will be homogeneous by design, we anticipate being able to legitimately pool the results. We are also aware that the underlying populations differ and we will therefore use a random effects model.

We will use Cochrane’s statistical software RevMan, and the statistical guidelines from the current version of the Cochrane Handbook will be followed ^12^. Due to the heterogeneity of the included trials we will use the random effects model. Although the included studies will be chosen for homogeneity of intervention and control, we will assess heterogeneity to confirm whether pooling data is legitimate.

We will adjust for clustering using intra-cluster coefficient estimates and average cluster sizes.

***Subgroup and sensitivity analyses.*** Evidence suggests that the effect of varying communication is likely to vary according to type of disease and type of outcome ^13, 14^. When the number of SWATs included in this meta-analysis permits, we will do separate analyses for different disease groups (for example, cancer, musculoskeletal, mental illness), population types (for example, children, adults), and intervention types (for example, psychological, pharmacological).

### Meta-bias(es)

To help determine whether there were meta-biases, we will investigate whether the outcomes in the meta-analysis were pre-specified in a protocol.

### Confidence in cumulative evidence

We will use the Grading of Recommendations Assessment, Development and Evaluation (GRADE) method to determine the confidence in cumulative evidence. This will involve assessment across all GRADE domains (risk of bias, consistency, directness, precision, and publication bias). The outcome of the GRADE assessment will be an overall assessment of the confidence in cumulative evidence as high, moderate, low, or very low.

## Amendments

In the event of protocol amendments, the date of each amendment will be accompanied by a description of the change and the rationale.

## Study status

This meta-analysis has not begun.

## Dissemination of results

The results will be disseminated through peer reviewed publication (a living meta-analysis) and conference presentations. Our systematic review protocol will be uploaded to ResearchGate.

## Ethics approval and consent to participate

Ethics approval is not required for this study since no original data will be gathered. Consent for participation is not relevant as there are no participants.

## Data Availability

No data are associated with this article. Harvard Dataverse: PRISMA-P checklist for ‘Patient reported outcomes and recruitment rates following the introduction of principled patient information leaflets (PrinciPILs): Protocol for a meta-analysis’. https://doi.org/10.7910/DVN/2IQO1H ^8^ Data are available under the terms of the Creative Commons Zero "No rights reserved" data waiver (CC0 1.0 Public domain dedication).
