# Supplementary material for: Patient reported outcomes and recruitment rates following the introduction of principled patient information leaflets (PrinciPILs): Protocol for a meta-analysis
Source: NIHR Open Res. 2023 May 26;3:29. [Version 1] doi: 10.3310/nihropenres.13420.1 (PMC11319896; doi:10.3310/nihropenres.13420.1)
Supplement: Peer review history

# Reviewer report: Reviewer response for version 1

**DOI:** 10.3310/nihropenres.14554.r31354

**Published:** 2024-05-16

**Authors:** Jing Kang

**Affiliations:** 1 King's College London (Ringgold ID: 4616), London, England, UK; 2 University of Leeds, Leeds, UK

This study protocol aims to assess the effects of recruitment rates and patient reported outcomes (including adverse outcomes) by comparing ‘principled patient information leaflet’ versus ‘standard patient information leaflet’.

 The rationale and background are clearly described, and the study design is appropriate for the research question. Overall, I feel this protocol is well designed and considered all possible biases and heterogeneity, and proposed solutions or assessments on those. The research idea is clinical meaningful and have good patient benefit implications.

 However, I have some major concerns:

1. The inclusion criteria are very board: no restriction of disease type, population, intervention methods, etc. Especially for the disease type, it wouldn’t be fair to pool all disease types and compare their recruitment rate, as rare disease and common disease are hardly comparable regardless of the outcome measures.
2. If the included number of trials are too large, how can the research team ensure completion of the project within the required timeframe?

Is the study design appropriate for the research question?

Yes

Is the rationale for, and objectives of, the study clearly described?

Yes

Are sufficient details of the methods provided to allow replication by others?

Yes

Are the datasets clearly presented in a useable and accessible format?

Not applicable

Reviewer Expertise:

medical statistics

I confirm that I have read this submission and believe that I have an appropriate level of expertise to confirm that it is of an acceptable scientific standard.

# Reviewer report: Reviewer response for version 1

**DOI:** 10.3310/nihropenres.14554.r31359

**Published:** 2024-04-01

**Authors:** Julie Bruce

**Affiliations:** 1 University of Warwick, Coventry, England, UK

This is a well written protocol from a leading team of trial methodologists. The systematic review aims to evaluate whether adapted patient information leaflets increase participant recruitment to clinical trials, reduce harms and improve quality of life compared to 'standard' PILS. 

 A few points for the team to consider:- 

1. It would be helpful to add some more background detail on the principles and adaptations made in the five host trials. Were the same team involved with adapting the PILs for all host trials thus there is confidence that all adapted PILs are indeed fully 'adapted' and whether/how this will be monitored when future studies are added. If this protocol will be stand alone for future SWATS to be added, what judgements will be made at screening stage. Whether all of the principles need to be adhered to to fulfil the definition of PrinciPIL. 
2. If you could check for consistency in relation to the description of outcomes across the protocol. The title states patient reported outcomes rather than patient-reported adverse events whereas the abstract states early effects on patient reported AEs. Under 'outcomes & prioritization' it specifies AEs, quality of life and mortality. Is the assumption here that the adapted PIL can reduce (or increase?) mortality? Also includes illness symptoms - but this could be any potential physical or psychological symptom thus the list could be very long. Whether to focus on symptoms with a potential mechanism of action e.g. anxiety, depression, catastrophizing etc. You do specify under subgroup analyses about disease groups but whether some classification is required for the illness symptoms/AEs. Are you only interested in patient-reported AE rather than site reported AEs- is there any consideration of expectedness? Or are all symptoms considered to be AEs. Will site-reported events be included?
3. Pain, patient satisfaction and retention are also mentioned under the eligibility criteria section.  Thus the terminology or listing of outcomes needs to be consistent across the protocol. Whether to broadly categorise as i) process-related (or whatever terminology) for recruitment and retention issues, ii) patient-reported for specific symptoms, patient satisfaction etc. 
4. Suggest add systematic review to title as may want to include future studies although they may not all contribute to the meta-analyses? Meta-analysis is the statistical technique for synthesis not study design (think is a PRISMA requirement).
5. If this is going to be a live ongoing review, will searches of bibliographic databases be conducted? Whether to add in a covering statement for future updates - obviously for this review you are starting with the five host trials but whether searches will be completed for updates. Thus just thinking about future-proofing your review protocol. Also assume any phase of trial would be eligible although assume all will probably be Phase III studies of healthcare interventions. 

Just some things to consider, but overall a really interesting project and good luck to the research team.

Is the study design appropriate for the research question?

Yes

Is the rationale for, and objectives of, the study clearly described?

Yes

Are sufficient details of the methods provided to allow replication by others?

Partly

Are the datasets clearly presented in a useable and accessible format?

Yes

Reviewer Expertise:

Clinical trials, rehabilitation research

I confirm that I have read this submission and believe that I have an appropriate level of expertise to confirm that it is of an acceptable scientific standard, however I have significant reservations, as outlined above.

# Reviewer report: Reviewer response for version 1

**DOI:** 10.3310/nihropenres.14554.r31360

**Published:** 2024-03-29

**Authors:** Marie Falahee

**Affiliations:** 1 University of Birmingham, Birmingham, England, UK

This protocol describes a living meta-analysis of studies within trials (SWATs) comparing standard patient information leaflets with versions that present information about benefits and harms in line with the principles developed in an MRC funded initiative to reduce variation in the presentation of such information to trial participants (PrinciPILs).

 The protocol is well-written, the methods outlined are appropriate to address the research objectives with evidence of patient and public involvement, and the approach described follows appropriate guidelines. My comments are minor, and largely concern the provision of additional detail.

 1. The introduction is very concise, and would benefit from more detailed exposition of the principles underlying the PrinciPILs, and how this differs from the standard approach.

 2. The authors plan to summarize the differences between the PILs and the PrinciPILs for each trial. Please clarify whether/how inter-trial variation in the extent of the difference between the intervention and the comparator will be addressed in the analysis.

 3. The authors note that future SWATs of PrinciPILs may be included in this living analysis. I assume this could possibly include SWATs undertaken by other research teams outside of the current project. If so please note how these will be identified.

 4. Have the authors considered undertaking qualitative evaluation to explore participant responses to PILs and PrinciPILs? Could such information be incorporated into this study? If so, how?

Is the study design appropriate for the research question?

Yes

Is the rationale for, and objectives of, the study clearly described?

Yes

Are sufficient details of the methods provided to allow replication by others?

Yes

Are the datasets clearly presented in a useable and accessible format?

Yes

Reviewer Expertise:

health psychology, patient preferences, mixed methods

I confirm that I have read this submission and believe that I have an appropriate level of expertise to confirm that it is of an acceptable scientific standard, however I have significant reservations, as outlined above.

# Reviewer report: Reviewer response for version 1

**DOI:** 10.3310/nihropenres.14554.r29368

**Published:** 2023-07-20

**Authors:** Meike C. Shedden-Mora

**Affiliations:** 1 Department of Psychology, Medical School Hamburg, Hamburg, Germany

This protocol describes the planned meta-analysis of studies within trials (SWATs) comparing standard patient information leaflets (PILs) with a new approach, the principled patient information leaflets (PrinciPILs). PrinciPILs aim to overcome the risks of standard PILs of eliciting nocebo effects by specifically presenting side effect information in an improved way.

 The protocol is generally really well described and precise. However, there are some issues that could help the reader to better understand the overall approach. Moreover, I have some methodological questions, as outlined below.

1. Introduction: It would really help the reader to provide some more information on the PrinciPILs, and on how they differ from standard PILs.
2. Information sources: If I understand correctly, the primary author of this protocol has been involved in all SWATs of PrinciPILs. As the authors are probably aware of the number of SWATs conducted, it would be helpful to state this number here (although normally not common in protocols).
3. Outcomes and prioritization: What is the primary outcome, what are the secondary outcomes? Will the primary outcome be overall adverse events or side effect burden reported in the PrinciPILs groups, compared to standard PILs? It would be helpful to be more precise.
4. Outcomes and prioritization: Nocebo effects are more pronounced in nonspecific side effects such as headaches, fatigue or dizziness, compared to specific side effects, (e.g., fever after vaccination). I suggest that you could consider to define nonspecific side effects or symptoms across the SWATs, compared to specific side effects. This might give you a clearer picture on how PrinciPILs can improve nocebo effects.
5. Data synthesis: I was wondering whether the authors have considered to perform patient level meta-analysis. If I understand correctly, the authors have all patient-level data at hand. This could provide a much clearer picture of potential nocebo effects.

Is the study design appropriate for the research question?

Yes

Is the rationale for, and objectives of, the study clearly described?

Yes

Are sufficient details of the methods provided to allow replication by others?

Yes

Are the datasets clearly presented in a useable and accessible format?

Not applicable

Reviewer Expertise:

placebo and nocebo effects, treatment expectations, persistent physical symptoms, somatic symptom disorder

I confirm that I have read this submission and believe that I have an appropriate level of expertise to confirm that it is of an acceptable scientific standard.
